# Use of and Satisfaction with Telemedicine Services during the Pandemic: Findings from the COVID-19 Snapshot Monitoring in Germany (COSMO)

**DOI:** 10.3390/healthcare10010092

**Published:** 2022-01-04

**Authors:** André Hajek, Freia De Bock, Christina Merkel, Benedikt Kretzler, Hans-Helmut König

**Affiliations:** 1 Hamburg Center for Health Economics, Department of Health Economics and Health Services Research, University Medical Center Hamburg-Eppendorf, 20246 Hamburg, Germany; b.kretzler.ext@uke.de (B.K.); h.koenig@uke.de (H.-H.K.); 2 Federal Centre for Health Education, 50825 Cologne, Germany; Freia.DeBock@bzga.de (F.D.B.); Christina.Merkel@bzga.de (C.M.)

**Keywords:** COVID-19, corona-virus, SARS-CoV-2, telemedicine, online consultations, telehealth, digital health

## Abstract

Our aim was to investigate to what extent physician visits were replaced by telemedicine services because of the COVID-19 pandemic and the satisfaction with such telemedicine services. Cross-sectional data from the “COVID-19 Snapshot Monitoring in Germany” (COSMO, wave 49 from 11 to 12 August 2021 with *n* = 967). The average age was 44.9 years (SD: 15.6 years, ranging from 18 to 74 years) and 50.8% were female. Indiviuals were asked whether any physician visit was replaced by a telemedicine service (e.g., video consultation) since March 2020 because of the pandemic (yes, once; yes, several times; yes, always; no, not replaced; no, there was no need to see a doctor). Additionally, individuals who gave positive responses (i.e., yes, once; yes, several times; yes, always) were asked how satisfied they were with the corresponding telemedicine services (from 1 = very dissatisfied to 7 = very satisfied). While 55.4% of the respondents reported no need to see a doctor and 31.3% of the respondents did not replace physician visits by telemedicine services, about 13.3% of the respondents did replace physician visits by telemedicine services (4.8%: yes, once; 6.4%: yes, several times; 2.1%: yes, always). Among the individuals who used such services, the average satisfaction was moderately high (4.7, SD: 2.0). Additionally, several correlates of the replacing telemedicine service use were identified (e.g., perceived severity of a COVID-19 infection). In conclusion, about one out of seven individuals replaced physician visits by telemedicine services during the pandemic. For example, knowledge about the correlates of satisfaction with such services might be of importance to increase the quality of such services.

## 1. Introduction

In general, telemedicine refers to the delivery of medical care and provision of general health services from a distance [1] (e.g., medical aftercare of patients after surgery via video). It has grown steadily in the past decades, also driven by increased greater availability of the internet and faster (high speed) connections [1]. By telemedicine, patient outcomes can be enriched as it improve access to care [1]. Moreover, telemedicine services can also contribute to work satisfaction in physicians [2]. The need for telemedicine is also increasing, among other things, for reasons of poor access of physicians (particularly in rural areas) and high economic burden for the healthcare system.

Telemedicine can also assist in avoiding social contacts during the COVID-19 pandemic, one of the main measures against virus spread [3]. This is especially relevant for individuals who have a high risk for a severe course of COVID-19 [4]. Thus, the use of telemedicine increased considerably in various countries during the pandemic [5]. For example, in Germany, 25% of doctors’ offices offered video consultations in March 2020, whereas only about 2% offered these services in 2017 [6]. Additionally, about 1.4 million hours of online consultations were performed in the first half of 2020 which reflects an extraordinarily increase compared to times prior to the pandemic [6]. Moreover, about 20% of adults stated that they used telemedicine services (telephone, video or both) instead of physician visits in Germany in December 2020. It should be noted that the study by Reitzle et al. [7] solely focused on the replacement of physician visits by telemedicine services in Germany in December 2020 (i.e., a snapshot from the situation—including satisfaction with such services—in December 2020), whereas our current study focused on replacement of physician visits by telemedicine services in Germany over the whole course of the pandemic (from March 2020 to August 2021). Another similar study only focused on patient satisfaction with telemedicine in a single-institution, urban, quaternary academic medical center in New York City until the end of May 2020, whereas we used data from the general adult population (and included later stages of the pandemic).

Thus far, there is limited knowledge regarding the total extent to which physician visits were replaced by telemedicine services because of the pandemic and the overall satisfaction with such telemedicine services in Germany. Consequently, our aim was to close this gap in knowledge. 

To better understand the pandemic (and the measures as well as use of healthcare services) in Germany, a short description is given: Nationwide actions to prevent the spread of COVID-19 started in mid-March 2020 (e.g., closing of schools). In mid-April 2020, some restrictions were eased. Schools were re-opened in May 2020. Further restrictions were loosened in the following months. Since a sharp increase of the infection rate was observed in autumn 2020, several restrictions were tightened. In May 2021, the restrictions were eased. With regard to use of healthcare services, for example elective surgery in hospitals was postponed in Germany since March 2020 [8]. Moreover, a sizable proportion of individuals postponed screening procedures [9,10,11]. However, the perceived access to healthcare services was quite good [12]. 

## 2. Materials and Methods

### 2.1. Sample

Cross-sectional data were derived from wave 49 of the COVID-19 Snapshot Monitoring (COSMO) [13]—a repeated cross-sectional survey. This wave was used for reasons of data availability. This means that the outcome measures reported were solely quantified in this wave (and thus not in previous waves). COSMO started in March 2020. At the beginning, a weekly online-survey was conducted (with about 1000 respondents in each wave). The study aimed to capture the broad psychosocial status of the German population during the pandemic. The time span between the waves increased over time. Thus, wave 49 took place from 10th to 11th August 2021 and comprised 967 participants. Participants were recruited by the online access panel of the market and social research company ‘respondi’ (and received remuneration for their participation). 

A quota-based sampling was applied. Based on an online-panel, individuals were drawn in a way that it reflects the gender, age (crossed-quota: gender × age) and federal state (uncrossed) in the German general population [14]. German speaking individuals who live in Germany (18 to 74 years) were included. 

Informed consent was obtained from all individual participants included in the study. Ethical approval for COSMO was obtained by University of Erfurt’s IRB (#202000302). All procedures performed in the COSMO studies involving human participants were in accordance with the ethical standards of the University of Erfurt institutional research committee and with the 1964 Helsinki Declaration and its later amendments or comparable ethical standards.

### 2.2. Dependent Variables

Individuals were asked whether any physician visit was replaced by a telemedicine service (e.g., video consultation) since March 2020 because of the corona situation. Answer options were: yes, once; yes, several times; yes, always; no, not replaced; no, there was no need to see a doctor. In logistic regression analysis, it was dichotomized (0 = no, not replaced; 1 = yes, once; yes, several times; yes, always). This also means that the answer option “no, there was no need to see a doctor” was not used in regression analysis. Thus, only individuals with a need to see a doctor were compared. 

Additionally, individuals who gave positive responses (i.e., yes, once; yes, several times; yes, always) were asked how satisfied they were with the corresponding telemedicine services. This 7-item Likert scale ranges from 1 = very satisfied to 7 = very dissatisfied (only the endpoints were labeled). For reasons of interpretability, this scale was reversed so that a higher value indicated a higher satisfaction with telemedicine services. 

### 2.3. Independent Variables

Several correlates were included in regression analysis. For example, socio-economic factors: Sex, age group (18 to 29 years; 30 to 49 years; 50 to 64 years; 65 years and over), migration background (no; yes), relationship/marriage (no; yes), educational level (up to 9 years; 10 years and more (without general qualification for university entrance); 10 years and more (with general qualification for university entrance)), children under 18 years (no; yes), profession in health care (no; yes), community size (≤5000 inhabitants; 5001–20,000 inhabitants; 20,001–100,000 inhabitants; 100,001–500,000 inhabitants; >500,000 inhabitants). Moreover, COVID-19 and health-related factors were included: perceived severity of a COVID-19 infection (from 1 to 7; higher values correspond to higher severity), perceived probability of a COVID-19 infection (from 1 = extremely unlikely to 7 = extremely likely), COVID-19 infection (no; yes) and having at least one chronic condition (no; yes). 

### 2.4. Statistical Analysis

First, sample characteristics were calculated. Thereafter, multiple logistic regressions were performed with replacement of physician visits by telemedicine services since March 2020 because of the corona situation (0 = no, not replaced; 1 = yes, once; yes, several times; yes, always) as outcome measure. 

Moreover, multiple linear regressions were performed with the satisfaction with such telemedicine services as outcome measures. It should be repeated that the satisfaction with such telemedicine services only refers to individuals who replaced physician visits by telemedicine services since March 2020 because of the corona situation.

The statistical significance was defined as *p* value of ≤0.05. Statistical analyses were performed using Stata 16.1 (Stata Corp., College Station, TX, USA).

## 3. Results

### 3.1. Sample Characteristics

**Figure 1:**
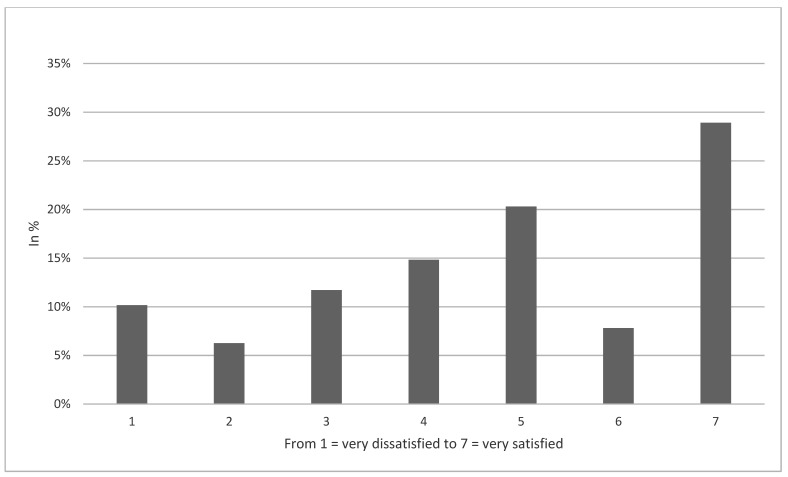
Satisfaction with the corresponding telemedicine services.

Sample characteristics are given in Table 1 (wave 49). Average age was 44.9 years (SD: 15.6 years, 18 to 74 years) and 50.8% were female. While 55.4% of the respondents reported no need to see a doctor and 31.3% of the respondents did not replace physician visits by telemedicine services since March 2020 because of the corona situation, about 13.3% of the respondents did replace physician visits by telemedicine services (4.8%: yes, once; 6.4%: yes, several times; 2.1%: yes, always). Among the individuals who used such services (as replacement of physician visits), the average satisfaction was moderately high (4.7, SD: 2.0). It is also displayed in a histogram (see Figure 1). Further details are given in Table 1. 

### 3.2. Regression Analysis

Multiple logistic regressions with replacement of physician visits by telemedicine services since March 2020 because of the corona situation (0 = no, not replaced; 1 = yes, once; yes, several times; yes, always) as outcome measure are displayed in Table 2. Regressions showed that the likelihood of replacement of physician visits by telemedicine services since March 2020 because of the corona situation was higher among individuals with low education (10 years and more (without general qualification for university entrance) compared to up to 9 years: OR = 0.37, 95% CI (0.15–0.92)), the presence of children under 18 years (OR = 1.98, 95% CI 1.12–3.50), higher perceived severity of a COVID-19 infection (OR = 1.23, 95% CI: 1.03–1.48) and already having had a COVID infection (OR = 3.58, 95% CI: 1.47–8.73). 

Based on multiple linear regressions, correlates of satisfaction with telemedicine services are displayed in Table 3. Among the individuals who used such services (as replacement), higher satisfaction with telemedicine services was associated with not having a migration background (β = −1.28, *p* < 0.01) and higher perceived severity of a COVID-19 infection (β = 0.36, *p* < 0.05), whereas the other potential correlates did not achieve statistical significance. 

## 4. Discussion

### 4.1. Main Findings

Based on data from the COSMO study, our aim was to investigate to what extent physician visits were replaced by telemedicine services because of the pandemic (over the course of the pandemic from March 2020 to August 2021) and the satisfaction with such telemedicine services during the whole pandemic. Our study showed that at more than one year after the beginning of the pandemic, about 13.3% of the respondents had replaced physician visits by telemedicine services over the course of the pandemic. Among the individuals who used such services, the average satisfaction was moderately high. Several correlates of using telemedicine as a replacement of personal visits were identified. 

### 4.2. Previous Research and Possible Explanations

The proportion of individuals replacing physician visits by telemedicine services in Germany during the pandemic mainly confirms previous findings made by Reitzle et al. (also conducted in Germany, but in December 2020) [7]. Additionally, they demonstrated that appointments were more frequently performed via telephone compared to video calls [7].

The moderately high satisfaction with such services may be explained as follows: Patients may perceive that such services sometimes cannot substitute, but supplement patient care [15]. Moreover, factors such as technical issues, internet connection problems or (online) waiting times may also explain why satisfaction with such services was not exceptionally high [16]. On the other side, individuals may truly appreciate the fact that they can at least supplement some physician visits via telemedicine services during times of the pandemic. In sum, our findings are also in line with the previously mentioned study in Germany (and international research, e.g., [17]) conducted during the pandemic [7]. 

Our study showed that the likelihood of replacement of physician visits by telemedicine services was higher among individuals with low education, individuals with children under 18 years, individuals with higher perceived severity of a COVID-19 infection and individuals already having had a COVID-19 infection. The association with a higher perceived severity of a COVID-19 infection appears plausible since these individuals may try their best to avoid physician visits (due to the fear of being infected). Moreover, individuals who already had a COVID-19 infection may have replaced physician visits by telemedicine services for quarantine/isolation reasons. Furthermore, individuals having children under 18 years may have multiple obligations during the pandemic (e.g., when kindergartens and schools were closed). Thus, telemedicine services may have mainly been used for reasons of time saving. The association between lower education and a higher likelihood of using telemedicine services (as replacement of physician visits) appears surprising and counterintuitive. Reitzle et al. also showed that video consultations were more often used by participants with a higher educational level [7]. A possible explanation may be that individuals with a lower education had worse possibilities for individual transport in pandemic (e.g., no own car and cannot affording a cab) compared to higher educated individuals. Thus, they may replace their physician visits, for example, with phone calls. However, future research is required to confirm our assumption. 

We showed that higher satisfaction with telemedicine services was associated with not having a migration background. One underlying, but speculative reason may be that individuals with migration background may have more difficulties with internet connectivity which may lower their satisfaction. Moreover, individuals with a higher perceived severity of a COVID-19 infection may particularly appreciate the opportunity to replace physician visits by telemedicine services (to avoid social contacts which may increase the likelihood of a COVID-19 infection). Thus, they may report higher satisfaction scores. In accordance with Reitzle et al., we did not identify sex differences in satisfaction with telemedicine services [7]. 

### 4.3. Strengths and Limitations

Several strengths and limitations have to be kept in mind. This is the first study quantifying the overall extent of replacement of physician visits by telemedicine services because of the pandemic (during the course of the pandemic from March 2020 to August 2021) and the satisfaction with such telemedicine services during the course of the pandemic in Germany. Data were used from the widely acknowledged COSMO study, with its limitations (no representativeness in terms of social status, selection bias/online-panel bias (e.g., individuals with poor access to the internet are commonly excluded) [18], and only including individuals up to 74 years). More precisely, in light of the recruitment process, the possibility cannot be entirely dismissed that the respondents of this survey are less afraid of using telemedicine services (than the general adult population in Germany) since these respondents are often used to dealing with the internet. Furthermore, small remunerations were given. It cannot be dismissed that this can affect the external validity of our study. 

Upcoming research should also examine the use of and satisfaction with telemedicine services among the oldest old. Moreover, future research is needed to quantify telemedicine services in further detail. Our current study solely focused on telemedicine service (e.g., video consultation). Other areas of telehealth were excluded. Furthermore, in accordance with previous research [19], a single item was used to assess satisfaction with telemedicine services. Future research based on more sophisticated tools is required to confirm our findings. Additionally, the present study has a cross-sectional design with the acknowledged limitations. Data are based on self-reports. Future research, e.g., based on claims data (if available) are required to confirm our findings. Furthermore, due to reasons of data availability, the specific types of replaced physician visits by telemedicine services (e.g., specific cases or situations) remain unknown. Thus, future research is required to clarify these conditions. 

## 5. Conclusions

About one in seven individuals replaced physician visits by telemedicine services since the pandemic. Additionally, the satisfaction with telemedicine services was moderately high. Moreover, knowledge about the correlates of satisfaction with such services (e.g., migration background) might be of importance to increase the quality of such services. To increase satisfaction, policy makers might consider the use of such information to specifically design telemedicine services for individuals with migration background (e.g., with automatic translation function).

## Figures and Tables

**Table 1:** Sample characteristics (*n* = 967).

Variables	Mean (SD)/*n* (%)
Age group	
- 18 to 29 years	200 (20.7%)
- 30 to 49 years	364 (37.6%)
- 50 to 64 years	251 (26.0%)
- 65 years and over	152 (15.7%)
Gender	
- Men	476 (49.2%)
- Women	491 (50.8%)
Migration background	
- No	811 (84.7%)
- Yes	146 (15.3%)
Relationship/Marriage	
- No	324 (33.5%)
- Yes	643 (66.5%)
Level of education	
- up to 9 years	104 (10.8%)
- 10 years and more (without general qualification for university entrance)	317 (32.8%)
- 10 years and more (with general qualification for university entrance)	546 (56.5%)
Children under 18 years	
- No	654 (67.6%)
- Yes	313 (32.4%)
Profession in health care	
- No	886 (91.6%)
- Yes	81 (8.4%)
Community size	
- ≤5000 inhabitants	153 (15.8%)
- 5001–20,000 inhabitants	228 (23.6%)
- 20,001–100,000 inhabitants	248 (25.6%)
- 100,001–500,000 inhabitants	173 (17.9%)
- >500,000 inhabitants	165 (17.1%)
Perceived severity: COVID-19 infection (from 1 to 7; higher values correspond to higher severity)	3.9 (1.5)
Perceived probability: COVID-19 infection (from 1 = extremely unlikely to 7 = extremely likely)	3.2 (1.4)
COVID-19 infection	
- No	911 (94.2%)
- Yes	56 (5.8%)
At least one chronic condition	
- No	585 (61.9%)
- Yes	360 (38.1%)
Replacement of any physician visits by a telemedicine service since March 2020 because of the corona situation	
- Yes, once	46 (4.8%)
- Yes, several times	62 (6.4%)
- Yes, always	20 (2.1%)
- No, not replaced	303 (31.3%)
- No, there was no need to see a doctor	536 (55.4%)
Satisfaction with the corresponding telemedicine services (from 1 = very dissatisfied to 7 = very satisfied)	4.7 (2.0)

Notes: Satisfaction with such telemedicine services only refers to individuals who replaced physician visits by telemedicine services since March 2020 because of the corona situation.

**Table 2:** Correlates of replacement of physician visits by telemedicine services since March 2020 because of the corona situation (0 = no, not replaced; 1 = yes, once; yes, several times; yes, always). Findings of multiple logistic regressions.

Independent Variables	Replacement of Physician Visits by Telemedicine Services Since March 2020 Because of the Corona Situation
Age group: - 30 to 49 years (Ref.: 18 to 29 years)	0.59
	(0.30–1.13)
- 50 to 64 years	0.50 +
	(0.24–1.06)
- 65 years and over	0.57
	(0.22–1.46)
Gender: Women (Ref.: Men)	0.93
	(0.58–1.50)
Migration background: Yes (Ref.: No)	0.75
	(0.39–1.44)
Relationship/Marriage: Yes (Ref.: No)	1.09
	(0.64–1.86)
Level of education: - 10 years and more (without general qualification for university entrance) (Ref.: up to 9 years)	0.37 *
	(0.15–0.92)
- 10 years and more (with general qualification for university entrance)	0.85
	(0.36–1.97)
Children under 18 years: Yes (Ref.: No)	1.98 *
	(1.12–3.50)
Profession in health care: Yes (Ref.: No)	1.33
	(0.58–3.08)
Community size: - 5001–20,000 inhabitants (Ref.: ≤5000 inhabitants)	1.60
	(0.69–3.73)
- 20,001–100,000 inhabitants	1.48
	(0.63–3.44)
- 100,001–500,000 inhabitants	1.95
	(0.81–4.69)
- >500,000 inhabitants	2.32 +
	(0.96–5.60)
Perceived severity: COVID-19 infection (from 1 to 7; higher values correspond to higher severity)	1.23 *
	(1.03–1.48)
Perceived probability: COVID-19 infection (from 1 = extremely unlikely to 7 = extremely likely)	1.21 +
	(1.00–1.47)
COVID-19 infection: Yes (Ref.: No)	3.58 **
	(1.47–8.73)
At least one chronic condition: Yes (Ref.: No)	1.25
	(0.76–2.08)
Constant	0.06 ***
	(0.01–0.27)
Observations	422
Pseudo R^2^	0.14

Notes: Odds ratios are reported, 95% confidence intervals in parentheses; *** *p* < 0.001, ** *p* < 0.01, * *p* < 0.05, + *p* < 0.10.

**Table 3:** Correlates of satisfaction with telemedicine services (from 1 = very dissatisfied to 7 = very satisfied). Findings of multiple linear regressions.

Independent Variables	Satisfaction with Telemedicine Services
Age group: - 30 to 49 years (Ref.: 18 to 29 years)	−0.10
	(0.46)
- 50 to 64 years	0.21
	(0.61)
- 65 years and over	0.84
	(0.70)
Gender: Women (Ref.: Men)	−0.01
	(0.41)
Migration background: Yes (Ref.: No)	−1.28 **
	(0.42)
Relationship/Marriage: Yes (Ref.: No)	0.80
	(0.49)
Level of education: - 10 years and more (without general qualification for university entrance) (Ref.: up to 9 years)	−0.06
	(0.78)
- 10 years and more (with general qualification for university entrance)	0.33
	(0.73)
Children under 18 years: Yes (Ref.: No)	−0.51
	(0.47)
Profession in health care: Yes (Ref.: No)	−0.51
	(0.52)
Community size: - 5001–20,000 inhabitants (Ref.: ≤5000 inhabitants)	0.37
	(0.62)
- 20,001–100,000 inhabitants	−0.22
	(0.63)
- 100,001–500,000 inhabitants	−0.06
	(0.66)
- >500,000 inhabitants	0.33
	(0.61)
Perceived severity: COVID-19 infection (from 1 to 7; higher values correspond to higher severity)	0.36 *
	(0.16)
Perceived probability: COVID-19 infection (from 1 = extremely unlikely to 7 = extremely likely)	−0.02
	(0.13)
COVID-19 infection: Yes (Ref.: No)	0.76
	(0.47)
At least one chronic condition: Yes (Ref.: No)	−0.11
	(0.42)
Constant	2.86 *
	(1.14)
Observations	124
R^2^	0.23

Notes: Unstandardized beta-coefficients are reported, robust standard errors in parentheses; ** *p* < 0.01, * *p* < 0.05; satisfaction with such telemedicine services only refers to individuals who replaced physician visits by telemedicine services since March 2020 because of the corona situation.

## Data Availability

The data from the COVID-19 Snapshot Monitoring (COSMO) are stored in the repository PsychArchives (https://doi.org/10.23668/psycharchives.2776 (accessed on 28 December 2021)) and are made available to scientists upon request. Requests should be submitted to the COSMO consortium by contacting Prof. Dr. Cornelia Betsch, University of Erfurt, Nordhäuser Str. 63, 99089 Erfurt, Germany (cornelia.betsch@unierfurt.de).
